# Outbreak of toxoplasmosis associated with muscular lesions in finishing pigs caused by an atypical *Toxoplasma gondii* genotype

**DOI:** 10.1590/S1984-29612022055

**Published:** 2022-10-28

**Authors:** Manoela Marchezan Piva, Paula Reis Pereira, Claiton Ismael Schwertz, Márcia Elisa Hammerschmitt, Marina Paula Lorenzett, Luan Cleber Henker, David Driemeier, Yara Souza Clemes, Hilda Fátima Jesus Pena, Saulo Petinatti Pavarini

**Affiliations:** 1 Setor de Patologia Veterinária, Faculdade de Veterinária, Universidade Federal do Rio Grande do Sul – UFRGS, Porto Alegre, RS, Brasil; 2 Departamento de Medicina Veterinária Preventiva e Saúde Animal, Faculdade de Medicina Veterinária e Zootecnia – FMVZ, Universidade de São Paulo – USP, São Paulo, SP, Brasil

**Keywords:** Swine diseases, Toxoplasma gondii, myositis, genotyping, PCR-RFLP, microsatellite analysis, Doenças dos suínos, Toxoplasma gondii, miosite, genotipagem, PCR-RFLP, análise de microssatélites

## Abstract

*Toxoplasma gondii* infections are usually asymptomatic in pigs, and an acute clinical disease is rare in this host. This study aimed to determine the pathological and molecular aspects of an outbreak of fatal systemic toxoplasmosis in finishing pigs in Brazil. The outbreak occurred on a commercial finishing pig farm in the state of Santa Catarina in southern Brazil. The farm had 1500 pigs and 3.8% of mortality rate during the outbreak. The pigs had fever, anorexia, apathy, and locomotor deficits. Seven pigs were necropsied. Gross findings included multifocal to coalescent pale areas in skeletal muscles, lymphadenomegaly, hepatosplenomegaly, and non-colapsed lungs. The histological findings included granulomatous lymphadenitis, hepatitis and splenitis, necrotizing myositis, and lymphoplasmacytic interstitial pneumonia. Lung and liver lesions were occasionally accompanied by *T. gondii* parasitic structures. Positive immunolabeling for *T. gondii* tachyzoites and encysted bradyzoites was detected in all examined pigs. PCR-RFLP (11 markers) and microsatellite analysis (15 markers) identified the non-archetypal genotype #278 in pigs. This is the first report of systemic toxoplasmosis in pigs with muscle lesions and additionally shows the diversity of disease-causing *T. gondii* genotypes circulating in animals in Brazil.

## Resumo

As infecções por *Toxoplasma gondii* são geralmente assintomáticas em suínos, e uma doença clínica aguda é rara nessa espécie. Este estudo teve como objetivo determinar os aspectos patológicos e moleculares de um surto de toxoplasmose sistêmica fatal em suínos em terminação no Brasil. O surto ocorreu em uma granja comercial de suínos em terminação no estado de Santa Catarina, no sul do Brasil. A granja tinha 1500 suínos e a taxa de mortalidade durante o surto foi de 3,8%. Os suínos apresentaram febre, anorexia, apatia e déficits locomotores. Sete suínos foram necropsiados. Os achados macroscópicos incluíram áreas pálidas multifocais a coalescentes nos músculos esqueléticos, linfadenomegalia, hepatoesplenomegalia e pulmões não colapsados. Os achados histológicos incluíram linfadenite, hepatite, esplenite granulomatosa e miosite necrosante, assim como pneumonia intersticial linfoplasmocítica. Lesões pulmonares e hepáticas foram ocasionalmente acompanhadas por estruturas parasitárias de *T. gondii.* A imunomarcação positiva para taquizoítos e bradizoítos encistados de *T. gondii* foi observada em todos os suínos examinados. PCR-RFLP (11 marcadores) e análise de microssatélites (15 marcadores) identificaram o genótipo não arquetípico #278 em suínos. Este é o primeiro relato de toxoplasmose sistêmica em suínos com lesões musculares e, adicionalmente, demonstra a diversidade de genótipos de *T. gondii* causadores de doenças circulantes em animais no Brasil.

## Introduction

Toxoplasmosis is a zoonotic disease caused by *Toxoplasma gondii*, an obligate intracellular protozoan. Domestic and wild cats are the definitive hosts that excrete oocysts into the environment, with other species of mammals, including humans, acting as intermediate hosts (Tenter et al., 2000). Infections occur after the ingestion of food or water contaminated with oocysts or through the consumption of uncooked or undercooked meat containing tissue cysts, which usually results in subclinical disease in immunocompetent hosts (Dubey & Jones, 2008; Dubey, 2010a; Dubey & Lappin, 2011).

Central and South America are major hotspots for *T. gondii* genetic diversity, with almost 200 genotypes having been identified in the region. In contrast, North America, Europe, and Asia have fewer circulating genotypes, with a predominance of classical types II and III (Shwab et al., 2014). This genetic diversity can be associated with more severe forms of human toxoplasmosis; however, this association is unclear in animal hosts (Carme et al., 2009). Pork consumption may be an important source of *T. gondii* infection in humans (Feitosa et al., 2014). Acute toxoplasmosis in pigs is rare, and cases have been reported only in neonates (Dubey et al., 1990; Thiptara et al., 2006) and weaners (Liao et al., 2006; Klein et al., 2010). This study aimed to present the pathological and molecular aspects of an outbreak of fatal toxoplasmosis with muscular lesions in a herd of growing-finishing pigs caused by an atypical *T. gondii* genotype infection.

## Material and Methods

### Farm description

The farm had approximately 280 sows and 1500 growing-finishing pigs. The grower-finishers were housed in shared pens (1 m^2^ per pig) in pig-sheds with sidewall curtains. The pens were separated by compact walls with a compact floor, pacifier-type drinking fountains that formed the water depth, and automatic feeders. There was no perimeter fence surrounding pig-sheds to prevent close contact between the housed pigs and other animals (domestic and wild). Domestic cats were raised by the producers and had free access to the swine sheds. All pigs were fed commercial diets, and the water provided to them was obtained from an artisanal well. The pigs were vaccinated against *Mycoplasma hyopneumoniae*, *Glaesserella parasuis* (autogenous vaccine), and porcine circovirus type 2 (PCV-2) (two doses at 15 and 35 days of age).

### Sampling and histopathology analysis

The outbreak of pig mortality occurred on a farm located in the municipality of Nova Veneza in Santa Catarina state (SC), in the southern region of Brazil (S-28.636908359871796, W-49.50109523771801). During the outbreak, one pig that died and six euthanized because of a poor prognosis were subjected to necropsy. Fragments of the main organs of the thoracic and abdominal cavities and samples of the skeletal muscles, brain, and spinal cord were collected. These fragments were fixed in 10% buffered formalin solution, routinely processed for histopathology, and stained with hematoxylin and eosin (HE). Additionally, fresh samples of several tissues were collected and stored at -20°C for subsequent molecular analysis.

### Immunohistochemistry

Immunohistochemistry (IHC) for *T. gondii* was performed on selected sections of the lymph nodes, skeletal muscle, brain, and lungs from all pigs (7/7). For antigen retrieval, samples were incubated for 10 min with a polyclonal antibody (VRMD, Pullman, WA, USA) at a 1:1000 dilution with 0.1% trypsin. A modified avidin-biotin-peroxidase complex method (LSAB Universal kit, Dako Cytomation, Glostrup, Denmark) was employed using 3-amino-9-tilcarbazoln (AEC, K3469, Dako Cytomation, Glostrup, Denmark) as the chromogen. Brain sections from a case of *T. gondii* encephalitis in a dog were used as positive controls as previously described by Nascimento et al. (2017). Sections of the skeletal muscle and lymph nodes from all cases were subjected to IHC anti- porcine circovirus type 2 (PCV2) with a polyclonal antibody, as previously described (Corrêa et al., 2007). Sections of lymph nodes from a pig with circoviruses were used as positive controls. Primary antibodies were replaced with a universal negative control serum (BioCare Medical, CA, USA) in selected sections as negative controls for both tests (*Toxoplasma gondii* and PCV-2).

### Molecular analyzes

DNA extraction from the tissues (lymph nodes, liver, lungs, spleen, muscle, or blood) of six pigs was performed using a Dneasy® Blood & Tissue commercial kit (Qiagen® Inc., USA) according to the manufacturer’s protocols. Polymerase chain reaction (PCR) amplification of *T. gondii* was performed as described by Homan et al. (2000), using a 529 base pair (bp) repeat element (REP529) fragment as a target; DNA from the RH *T. gondii* reference strain was used as a positive control. The amplified DNA was visualized by electrophoresis on 2% agarose gels stained with SYBR^®^ Safe DNA gel stain (Invitrogen, USA). Negative controls (ultrapure water) were included in all PCR reactions.

Three of the four *T. gondii* positive PCR samples were then genotyped by multilocus PCR Restriction Fragment Length Polymorphism (PCR-RFLP), compared and classified according to other previously characterized *T. gondii* Brazilian strains available in the ToxoDB database (http://toxodb.org/toxo/) and recent publications.

PCR-RFLP was performed as described by Su et al. (2010), using the genetic markers SAG1, SAG2 (3’5’SAG2 and alt. SAG2), SAG3, BTUB, GRA6, C22-8, C29-2, L358, PK1, Apico (Su et al., 2006), and CS3 (Pena et al., 2008). Reference archetypal strains RH (Type I), PTG (Type II), and CTG (Type III) and *T. gondii* non-archetypal strains (TgCgCa1, MAS, and TgCatBr5) were included as positive controls and ultrapure water was used as negative control in all reactions. To refine the genotyping results, microsatellite analysis (MS) using 15 markers (TUB2, W35, TgMA, B18, B17, M33, IV.1, X1.1, N60, N82, AA, N61, N83, M48, and M102) was performed according to the protocol described by Ajzenberg et al. (2010), and the results were analyzed using Genemapper 4.1® (Applied Biosystems, Waltham, MA, USA). The classical Type II reference strain ME-49 was used as positive control and ultrapure water was used as negative control in all reactions.

This study was registered in the Sistema Nacional de Gestão do Patrimônio Genético e do Conhecimento Tradicional Associado (SisGen; idenfication number: A3C5173).

## Results

A pig farm owner contacted the Setor de Patologia Veterinária at the Universidade Federal do Rio Grande do Sul, after observing a significant increase in pig mortality in February 2020. A disease with a sudden onset was observed, affecting previously healthy grower-finishers. The affected pigs had anorexia, respiratory distress, and locomotion deficits characterized by muscle tremors, the inability to stand, or lateral recumbency, sometimes accompanied by a fever of up to 41.5ºC. The clinical course from the onset of clinical signs to death ranged from one to three days. Two disease peaks were also observed. In the first case peak (February 2020), 30 pigs died. One of these pigs was subjected to a postmortem examination by a field veterinarian, and tissue samples were submitted for histopathology. The microscopic findings in this case included interstitial pneumonia, interstitial nephritis, histiocytic and necrotizing hepatitis, and lymphadenitis. Based on these findings, a presumptive diagnosis of porcine circovirus-associated disease was established. The second peak occurred 30 days later (March 2020), in which 27 pigs died within three days. A total of 57 grower-finishers (57/1500) died in the first and second peaks of the disease, resulting in a mortality rate of 3.8%. During the second peak, an on-site visit to the pig farm was conducted to investigate the outbreak and collect samples.

**Figure 1:**
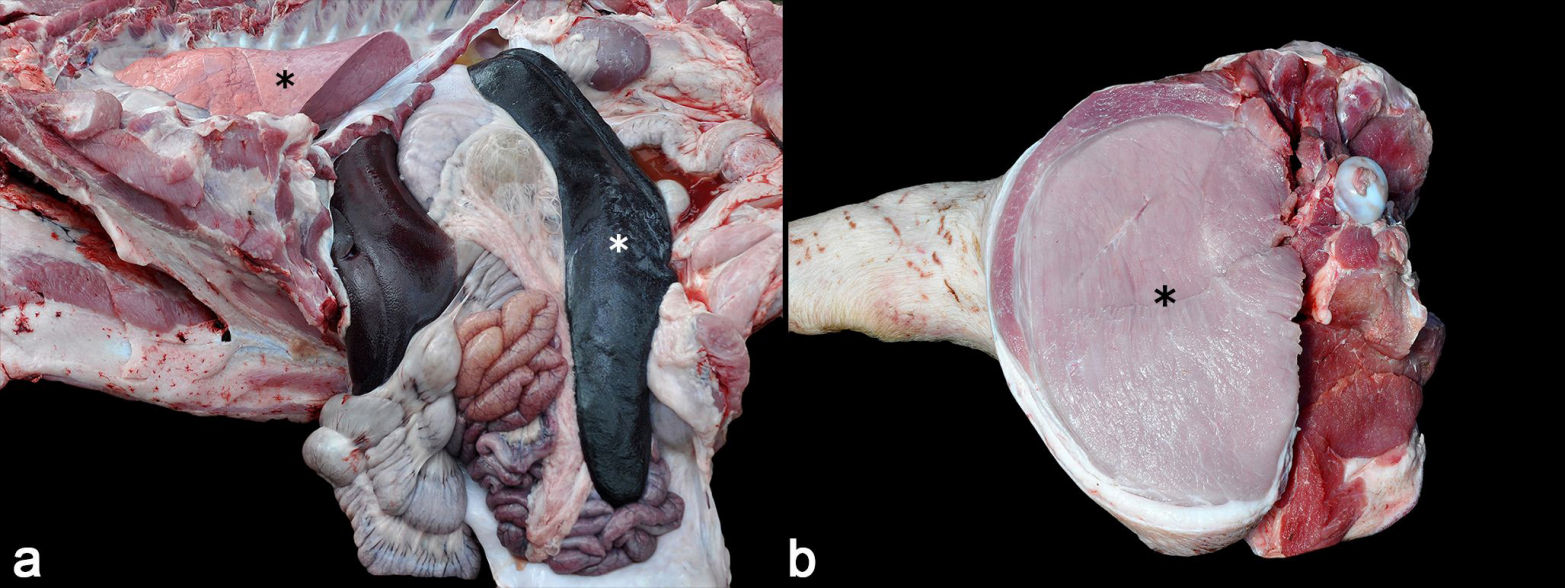
Macroscopic lesions of systemic toxoplasmosis in finishing pigs in Brazil. **a.** Abdominal and thoracic cavities. There is marked splenomegaly (white asterisk), and the lungs failed to collapse upon opening of the thoracic cavity (black asterisk). **b.** Skeletal musculature of the right pelvic limb, cut section. *Psoas major* and *psoas minor* muscle groups show diffuse and marked pale discoloration in comparison to the adjacent unaffected muscles (asterisk).

During the on-site visit, six grower-finishers pigs aged 102-135 days were euthanized and submitted to postmortem examinations. Gross findings included marked enophthalmos (dehydration) (4/6) and skin abrasions on the lateral aspect of the pelvic limbs (2/6). All pigs had markedly enlarged lymph nodes (generalized lymphadenomegaly), which were more prominent in the mesenteric and internal iliac lymph nodes (6/6). On the cut surface, the nodal parenchyma was nearly effaced by white tissue with a loss of corticomedullary differentiation. Mild to marked splenomegaly (Figure 1A) and mild ascites were observed in 5/6 and 4/6 cases, respectively. Liver enlargement was noted, accompanied by random multifocal white spots < 2 mm on the capsule surface and on the cut section (4/6). Mild kidney enlargement with multifocal to coalescing white areas < 2 mm on the surface and the cut sections were also seen (3/6). In four cases, mild to severe and focally extensive to diffuse areas of pale discoloration were observed in the skeletal musculature. These areas were more evident in the muscles of the pelvic limbs, *psoas major,* and *psoas minor* (4/6) (Figure 1B).

**Figure 2:**
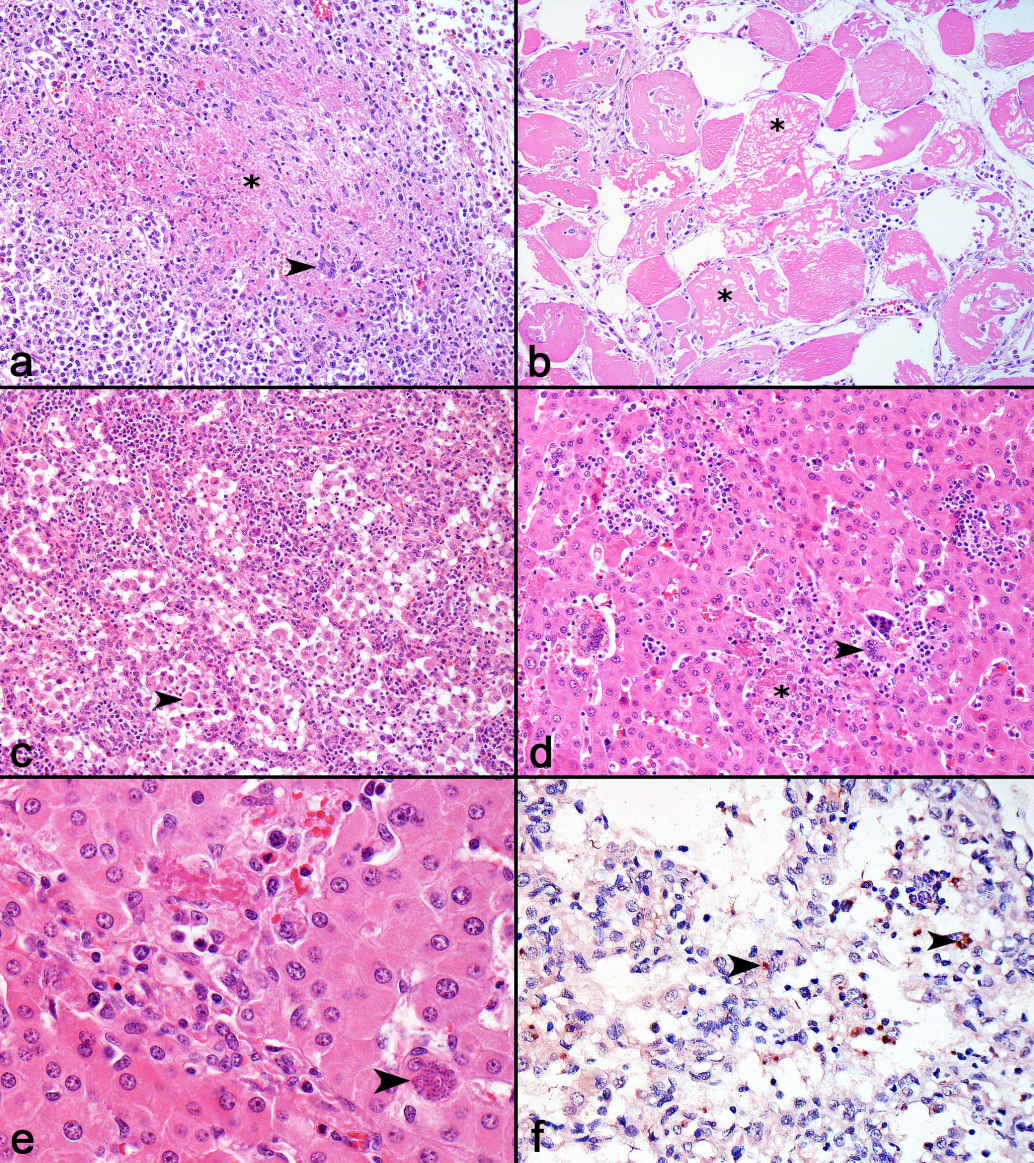
Microscopic lesions of systemic toxoplasmosis in finishing pigs in Brazil. **a.** Lymph node. There is an area of marked necrosis (asterisk), with intense fibrin deposition, accompanied by degenerate neutrophils, lymphocytes, plasma cells, macrophages, and multinucleated giant ells (head arrow). HE. 200x. **b.** Skeletal muscle. Marked and diffuse muscle necrosis, characterized by hypereosinophilic fibers with loss of striation, and cytoplasmic fragmentation (asterisk). Mild interstitial infiltrate of lymphocytes, plasma cells, macrophages and satellite cells are observed. Mild influx of macrophages in the cytoplasm of necrotic myocytes is also seen. HE. 200x. **c.** Lung. Alveolar septa are markedly thickened due to proliferation of type II pneumocytes and interstitial infiltrate. Alveolar spaces are filled with large numbers of free type II pneumocytes and syncytial cells (head arrow). HE. 200x. **d.** Liver. There are random foci of coagulative necrosis (asterisk), with inflammatory infiltrate of lymphocytes, plasma cells, macrophages, and multinucleated giant cells (head arrow), sometimes accompanied by moderate fibrin deposition. HE. 200x. **e.** Liver. Focal area of mild necrosis and fibrin deposition, accompanied by inflammatory infiltrate of lymphocytes, plasma cells, and fewer eosinophils. Adjacent to this area, there is a *T. gondii* cyst within the cytoplasm of a hepatocyte. This parasitic structure is morphologically compatible with encysted bradyzoites, measures approximately 25µm (cyst), is well delimited by a thin capsule, and contains small eosinophilic nuclei (head arrow). HE. 400x. **f**. Lung. Moderate multifocal immunolabeling of *T. gondii* tachyzoites, observed freely or within the cytoplasm of pneumocytes (1-3 µm), and of cysts (10-15 µm) (head arrows). IHC. AEC chromogen. 400x.

Histopathological findings were compiled for all cases; tissue slides from the first case submitted prior to the on-site visit were revised and assessed in conjunction with the six cases examined during the field visit. Microscopic findings included multifocal to coalescing areas of marked coagulative necrosis in the lymph nodes with intense fibrin deposition, moderate infiltration of neutrophils and macrophages, and variable numbers of multinucleated giant cells (7/7) (Figure 2A). Muscle lesions were characterized by multifocal areas with multiple hypereosinophilic swollen muscle fibers with a loss of cytoplasmic striations (hyaline necrosis) and cytoplasmic fragmentation (flocculate necrosis) (Figure 2B). Affected muscle fibers were surrounded by, and sometimes contained, a cytoplasmic influx of satellite cells and mild inflammatory infiltrate of lymphocytes, plasma cells, and macrophages (6/7), and fewer multinucleated giant cells (1/7). Less commonly, necrotic myocytes had cytoplasmic mineralization, and the affected areas had regenerated muscle fibers (1/7).

Lung lesions were characterized by mild to moderate diffuse interstitial pneumonia with an inflammatory infiltrate composed predominantly of lymphocytes and plasma cells (7/7), accompanied by intense proliferation of type II pneumocytes (7/7) and syncytial cells (3/7). Other lesions in the lungs included multifocal areas of mild necrosis, with a central area containing neutrophils and fibrin deposition, often accompanied by macrophages and multinucleated giant cells (5/7) and multifocal thrombosis (2/7) (Figure 2C). Multifocal, mild to moderate histiocytic splenitis was observed in 6/7 cases, sometimes accompanied by multifocal areas of mild necrosis (3/7) and multinucleated giant cells (1/7).

Random foci of coagulative necrosis forming small nodules were observed in the hepatic parenchyma in all cases (7/7). These areas were accompanied by fibrin deposition and inflammatory infiltration of neutrophils, lymphocytes, and macrophages, fewer eosinophils, and multinucleated giant cells (Figure 2D). Similar necrotic foci were also seen in the adrenal glands (1/7) and pancreas, accompanied by moderate peripancreatic fat necrosis (3/7). Multifocal to coalescing moderate lymphoplasmacytic interstitial nephritis was observed in 4/7 cases. Central nervous system lesions were present in three cases and were characterized by mild multifocal perivascular lymphoplasmacytic infiltrates in the leptomeninges and parenchyma (3/7), and mild multifocal areas of microgliosis, especially in the white matter of the telencephalon (2/7).

In 4/7 cases, rare small, and rounded structures measuring 15-30 µm and filled with basophilic granules (encysted bradyzoites) were observed in areas of necrosis, mainly within the cytoplasm of type II pneumocytes and macrophages in the lung, and in macrophages in the liver (Figure 2E). In these cases, oval or round structures measuring–3-5 µm, consistent with free tachyzoites, were observed in areas of inflammation and necrosis. *T. gondii* structures were not observed in histological sections of the remaining tissues.

Positive immunolabeling for *T. gondii* was observed in the tissue sections from all pigs (7/7). Positive immunolabeling was detected in sections of the lymph nodes (7/7), lungs (7/7), and brain (3/7). Granular immunolabeling of parasitic structures compatible with free tachyzoites was observed and was less frequent with encysted bradyzoites amid areas of necrosis and inflammation (Figure 2F) in sections of lymph nodes, lungs, and amid foci of gliosis in the brain. Encysted bradyzoites were more readily detected in the lung sections. No positive immunolabeling was detected in skeletal muscle tissue sections. All the cases were negative for IHC anti-PCV-2.

PCR for *T. gondii* was positive in four of the six cases examined (case 1: spleen; case 4: mesenteric lymph node; case 5: lungs; case 6: mesenteric lymph node). Genotyping was performed on three samples (spleen was not included in the study because DNA showed a very weak band in agarose gel using 529REP target). The strains were designated PS-TgPigBrSC1, PS-TgPigBrSC2, and PS-TgPigBrSC3 (PS, primary sample). The three strains were genotypically identical and corresponded to the atypical genotype PCR-RFLP #278 previously described by Pena et al. (2018) in a chicken isolate (TgCkBrSC4) from SC in Brazil (Table 1). Using MS, the three strains showed the same 15-allele profile, corresponding to a non-archetypal genotype and identical to the MS genotype from TgCkBrSC4 isolate (Table 2).

**Table 1:** Multilocus genotyping of *Toxoplasma gondii* by PCR - Restriction Fragment Length Polymorphism (RFLP) from pig samples during a toxoplasmosis outbreak.

			RFLP markers												PCR-
Strains	Municipality	State	SAG1	5´3´SAG2	alt. SAG2	SAG3	BTUB	GRA6	c22-8	c29-2	L358	PK1	Apico	CS3	RFLP genotype
PS-TgPigBrSC1,2,3	Nova Veneza	Santa Catarina	I	I	I	III	III	III	III	III	I	I	I	I	#278*

* The same genotype was identified in an isolate from chicken (TgCkBrSC4) from Florianópolis, Santa Catarina state by Pena et al. (2018).

**Table 2:** Multilocus genotyping of *Toxoplasma gondii* by microsatellite analysis (MS) from pig samples during a toxoplasmosis outbreak.

			Microsatellite markers															
Strains	Municipality	State	TUB2	W35	TgM-A	B18	B17	M33	MIV.1	MXI.1	M48	M102	N60	N82	AA	N61	N83	MS genotype
PS-TgPigBrSC1, 2,3	Nova Veneza	Santa Catarina	289	248	205	160	336	165	278	356	213	166	147	109	265	87	306	Non-archetypal*

* This genotype is a combination of alleles I/III, II/III, and III.

The same genotype was identified in an isolate from chicken (TgCkBrSC4) from Florianópolis, Santa Catarina state by Pena et al. (2018).

## Discussion

The diagnosis of systemic toxoplasmosis in this study was based on clinical, gross, and histopathological findings, with confirmation by immunohistochemical and molecular results. The use of histopathology alone to diagnose similar cases may prove difficult because parasites are not always readily detected in tissue sections. Therefore, immunohistochemistry and molecular assays are valuable tools for confirming toxoplasmosis diagnoses (Jones et al., 2012; Casagrande et al., 2015).

Generally, pigs infected with *T. gondii* are asymptomatic (Dubey, 2010b). Several factors may be associated with clinical manifestations and death in pigs due to systemic toxoplasmosis, including the virulence of the strain, dose, and individual and environmental factors (Dubey, 2010a). The origin of the toxoplasmosis was not investigated in this study. All infections occurred in grower-finishers, and cats had unrestricted access to the farm. Thus, food or water contaminated with *T. gondii* oocysts may have been the source of infection in pigs.

A non-archetypal genotype (PCR-RFLP #278) was identified by PCR-RFLP in this outbreak. This genotype is characterized by a combination of typical alleles I and III and was previously detected in an isolate from a free-range chicken (TgCkBrSC4), also from SC in Brazil (Pena et al., 2018); this isolate caused 100% of mortality in mice, but there is no direct association between virulence of strains in mice and other hosts, particularly because high virulence in mice seems to be a marked biological trait from strains isolated in Brazil (Pena et al., 2008). MS is a high-resolution tool useful to identify a common source of infection in an outbreak (Ajzenberg et al., 2010). Herein, although the exact source could not be established, it was confirmed that the animals were infected not only by a close strain, but also by the same non-archetypal lineage. This was the same lineage detected in the isolate TgCkBrSC4, suggesting a high circulation of this possible clone in the region, which is a risk factor for public health.

Although there are approximately 700 strains genotyped by PCR-RFLP from different domestic and wild animals and humans in Brazil (H.F.J.P, personal communication), information on *T. gondii* diversity is limited in SC. There are currently 14 genotyped strains in this state, which are classical clonal types I and II, and non-archetypal genotypes, including samples from cattle (Macedo et al., 2012), chickens (Trevisani et al., 2017 ; Pena et al., 2018;), and cats (Pena et al., 2017). Therefore, this study adds to what is currently known about *T. gondii* diversity in this Brazilian state by reporting *T. gondii* infection in a different host.

Most descriptions of *T. gondii* genotypes identified in swine samples in Brazil are from the northeastern region of Brazil (Olinda et al., 2016; Melo et al., 2020). Olinda et al. (2016) reported an outbreak of toxoplasmosis in swine associated with another non-archetypal genotype (RFLP #9 or Chinese 1). Additionally, Paraboni et al. (2020) described a new *T. gondii* non-archetypal genotype detected in pork samples from the state of Rio Grande do Sul in the southern region of Brazil.

Toxoplasmosis outbreaks with muscular lesions are uncommon in veterinary medicine; to the best of our knowledge, similar lesions have never been reported in pigs. Interestingly, no parasitic structures were observed in histological sections of the affected skeletal muscles. In humans, more information is available on muscle lesions associated with toxoplasmosis. Hassene et al. (2008) discussed cases of muscle damage evidenced by biochemical tests and biopsies in human patients who were seropositive for *T. gondii*. In that study, *T. gondii* was not identified through histopathology or PCR of muscle samples, similarly to that observed in this study. In this case, muscle necrosis may have been induced by an immunologic complication of toxoplasmosis associated with an immune complex-mediated systemic disease (Quilis & Damjanov, 1982). As this mechanism of injury is not well understood in animals, we were unable to establish the pathogenesis of the lesion.

An association between *T. gondii* seropositivity and increased muscle enzyme activity has been described in pigs, suggesting muscle injury, especially in sows with potentially compromised or suppressed immunity (Athanasiou et al., 2021). Although clinical toxoplasmosis in animals and humans is classically associated with immunosuppression, no signs of co-infection with PCV-2, an important immunosuppressive virus in pigs, have been found. Other diseases that cause systemic immunosuppression are classical swine fever and swine reproductive respiratory syndrome; however, the swine herd in southern Brazil is currently negative for these diseases.

Muscular lesions represented grossly by areas of pale discoloration, similar to those seen in our cases, have been previously reported in cases of granulomatous necrotizing myositis caused by PCV-2 infections (Konradt et al., 2018). Macroscopically, pigs affected by PCV-2-associated granulomatous myositis also had lymphadenopathy and interstitial nephritis, similar to the lesions found in this outbreak of toxoplasmosis in pigs. The similarities shared between these conditions led to a misdiagnosis in the first histopathological analysis. Although grossly indistinguishable, the muscular lesions associated with PCV-2 infections (Konradt et al., 2018) are richer in inflammatory cells and vasculitis, unlike the lesions displayed by pigs with toxoplasmosis *T. gondii* myositis. Lesions in these cases are predominantly necrotic, there is little association with inflammation, and there is no vasculitis, which is a special feature of circovirosis.

Toxoplasmosis in finishing pigs can occur as a feverish and fatal systemic disease and is noticeable owing to serious skeletal muscle injuries and clinical manifestations of locomotor deficits. To the best of our knowledge, similar necrotizing muscle lesions have not been previously associated with toxoplasmosis in pigs. Pathological findings characterized by systemic granulomatous and necrotizing lesions share similarities with PCV-2 infections, which appears to be the main differential diagnosis in similar cases. This study also contributes to the expanding knowledge on the diversity of *T. gondii* genotypes which circulate and cause clinical diseases in animal hosts in Brazil.

## Ethics declaration

The project that gave rise to the present data was approved to the Research Committee (COMPESQ) of the Universidade Federal do Rio Grande do Sul (UFRGS) (Project number 40376).

## Conflit of Interest

The authors declared no potential conflicts of interest with respect to the research, authorship or publication of this article.
